# Thermoresponsive Amphiphilic Polymer Emulsifier for Low-Dosage, High-Stability Oil-Based Drilling Fluids

**DOI:** 10.1021/acsomega.6c08519

**Published:** 2026-09-30

**Authors:** Dapeng Zou, Jun Wang

**Affiliations:** † College of Chemistry and Chemical Engineering, Northeast Petroleum University, Daqing 163318, China; ‡ Drilling Fluid Company, Daqing Drilling & Exploration Engineering Corporation, Daqing 163413, China

## Abstract

Reducing the oil-to-water ratio (OWR) in oil-based drilling fluids (OBDFs) offers significant economic and environmental benefits, yet severely challenges emulsion stability at high temperatures. To address this issue, we designed a thermoresponsive amphiphilic copolymer that dynamically reinforces the oil–water interface upon heating, thereby stabilizing low-OWR emulsions at elevated temperatures. Here, we report a thermoresponsive amphiphilic copolymer, poly­(St-NIPAM-AMPS) (PSNA), synthesized via free-radical polymerization, as a primary emulsifier for low-OWR OBDFs. PSNA incorporates styrene (St) as a hydrophobic anchor, *N*-isopropylacrylamide (NIPAM) as a thermoresponsive unit, and 2-acrylamido-2-methyl-1-propanesulfonic acid (AMPS) as an electrostatic stabilizer. Systematic optimization identified PSNA-2 (St:NIPAM:AMPS = 1:1:1) as the optimal composition. At an emulsifier loading of 6 wt % and an OWR of 70:30, the PSNA-2-based OBDF achieved an electrical stability of 879 V, a high-temperature, high-pressure filtration loss of 3.2 mL, and a sag factor of 0.513 after dynamic aging at 180 °C for 16 h, meeting all industry criteria. Mechanistic studies reveal a thermally triggered interfacial self-reinforcement mechanism: above its lower critical solution temperature (54 °C), the NIPAM segments collapse, densifying the interfacial film, while the AMPS groups provide sustained electrostatic repulsion. The PSNA-2 formulation also exhibits a nontoxic environmental profile (EC_50_ > 30,000 mg/L). This work offers a scalable, cost-effective emulsifier design for high-performance, low-OWR OBDFs.

## Graphical abstract

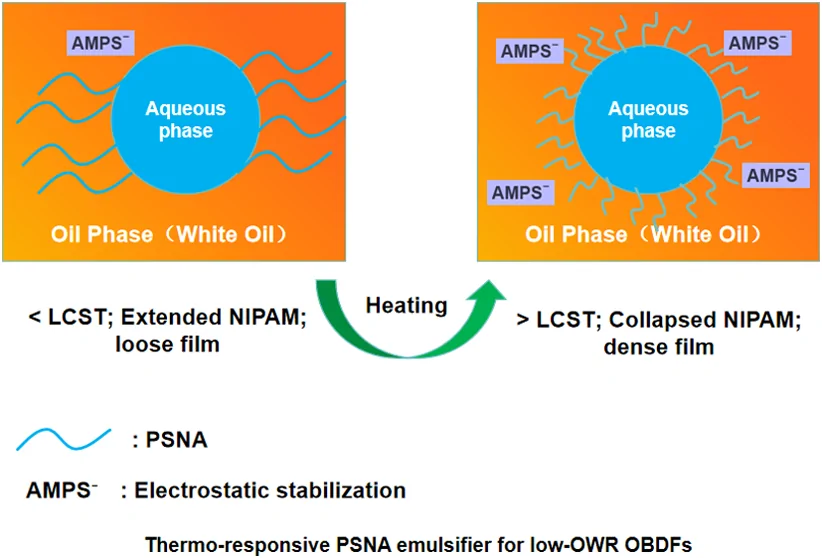

## 1 Introduction

Drilling fluids, often referred to as the “blood” of the drilling operation, serve multiple critical functions: they cool and lubricate the drill bit, transport cuttings to the surface, control formation pressure, maintain wellbore stability, and prevent formation damage. Oil-based drilling fluids (OBDFs) are preferred in such demanding conditions because they offer excellent lubrication, shale inhibition, thermal stability, and resistance to salt and calcium contamination.^1–5^ Deep and deviated wells impose severe challenges to wellbore stability and formation integrity, requiring drilling fluids that can maintain performance under extreme geomechanical conditions.^6^ Recent advances in environmentally benign polymer additives have demonstrated that sustainable materials can achieve high performance without compromising environmental compatibility.^7^ However, maintaining consistent performance at elevated temperatures and pressures remains challenging.^8^ A practical strategy to reduce both cost and environmental footprint is to lower the oil-to-water ratio (OWR). Reducing the OWR directly decreases the consumption of expensive synthetic or diesel-based base oils, which are the primary nonbiodegradable and environmentally persistent components of OBDFs. This reduction not only lowers the overall carbon footprint of the drilling operation but also minimizes the volume of oily cuttings and waste fluids requiring treatment and disposal. While conventional OBDFs typically operate at an OWR of 80:20 to 90:10, reducing the OWR to 70:30 or lower can substantially reduce base oil consumption. Nevertheless, such a reduction dramatically increases the oil–water interfacial area, placing severe demands on the emulsifier to form a dense, mechanically robust interfacial film that can withstand high temperatures and shear. Under these harsh conditions, conventional emulsifiers often suffer from thermal degradation, desorption from the interface, and film collapse, leading to emulsion instability, Barite sag, and excessive filtration loss.^9–11^

Considerable efforts have been devoted to developing improved emulsifiers, including polymeric surfactants, pH-sensitive reversible emulsifiers, hyperbranched polymers, and nanoparticle-assisted systems,^12–19^ and other advanced polymeric emulsifiers.^20,21^ For example, Li Lei et al.^18^ confirmed that CTAB is a reliable emulsifier for acidic emulsions, with rheological properties tunable by temperature and pH. However, CTAB and most conventional emulsifiers cannot dynamically reinforce the oil–water interface under heat. While pH-responsive, nanoparticle-assisted, and other “smart” emulsifier systems offer tunable properties under specific stimuli, most share a common limitation: they cannot dynamically and spontaneously reinforce the oil–water interface in response to the increasing thermal stress encountered during downhole circulation. Thermoresponsive polymers that undergo a coil-to-globule transition above their lower critical solution temperature (LCST) offer a promising design paradigm to address this challenge. PNIPAM is a well-known thermoresponsive polymer with an LCST around 32 °C in water,^22–24^ and its LCST can be readily tuned through copolymerization with hydrophobic or hydrophilic comonomers.^25,26^ The broader family of thermoresponsive polymers has demonstrated significant potential in adaptive interfacial materials.^27^ Beyond thermoresponsive systems, recent advances in sustainable polymer design—such as lignin-based polyurethane elastomers with dynamic cross-linking—have further underscored the versatility of stimuli-responsive and environmentally conscious polymer architectures.^28^ Beyond polymeric systems, the incorporation of waste-derived and functional composite materials has emerged as a promising approach for developing adaptive engineering materials.^29^ When incorporated into an amphiphilic copolymer, the NIPAM segments remain hydrated and extended at ambient temperature, facilitating initial emulsification. Upon heating to reservoir temperature (typically >100 °C), the NIPAM chains dehydrate and collapse, becoming hydrophobic. This conformational transition can drive the polymer chains to pack more densely at the oil–water interface, thereby creating a thermally triggered self-reinforcing interfacial film-highly desirable for low-OWR OBDFs under high-temperature conditions.

Herein, we report a thermoresponsive amphiphilic copolymer, PSNA, designed specifically as a primary emulsifier for low-OWR, high-density OBDFs. The copolymer incorporates three functional components: styrene St as a hydrophobic anchor, NIPAM as the thermoresponsive unit, and AMPS as a strongly ionizable sulfonate group that provides electrostatic stabilization and salt tolerance. PSNA is synthesized via a straightforward free-radical solution polymerization, which is cost-effective and scalable. Through systematic optimization of the monomer composition, emulsifier concentration, and OWR, the optimal PSNA-2 formulation (St:NIPAM:AMPS = 1:1:1) was identified. This formulation enables stable OBDF operation at a reduced OWR of 70:30 after dynamic aging at 180 °C, meeting all industry-critical criteria for emulsion stability, filtration control, and sag resistance. Comprehensive mechanistic characterization reveals a thermally triggered interfacial self-reinforcement mechanism: below the LCST, PSNA chains adopt an extended conformation, forming a loose interfacial layer; above the LCST (i.e., under downhole conditions), the NIPAM segments collapse, densifying and strengthening the film. Combined with sustained electrostatic repulsion from AMPS groups, this adaptive behavior counteracts the increased interfacial stress inherent to low-OWR formulations. This work presents a promising and scalable design strategy for adaptive thermoresponsive polymer emulsifiers, offering a simple, cost-effective, and environmentally benign pathway toward high-performance low-OWR OBDFs (EC_50_ > 30,000 mg/L). The thermoresponsive self-reinforcement strategy presented in this work addresses a critical gap in existing emulsifier technologies by exploiting heat—an unavoidable downhole condition—as an activating stimulus, offering a distinct advantage over conventional and alternative smart emulsifier systems that rely on external triggers such as pH adjustment or nanoparticle addition.

## 2 Materials and Methods

### 2.1 Materials

The experimental reagents and instruments used in this work are listed in Tables 1 and 2.

**Table 1:** Experimental Reagents^a^

Material	Purity/Grade	Supplier
Styrene (St)	95.0%	Wuhan Hengjiu Chemical Co., Ltd.
*N*-isopropylacrylamide (NIPAM)	99.0%	Shandong Hongyang Chemical Co., Ltd.
2-Acryloylamino-2-methyl-1-propanesulfonic acid (AMPS)	99.0%	Hubei Baidu Chemical Co., Ltd.
*p*-Toluenesulfonic acid	98.0%	Aladdin, Shanghai, China
Calcium hydroxide	99.0%	Aladdin, Shanghai, China
Oxidized asphalt	99.0%	Shandong Tonglan Chemical Co., Ltd.
Organobentonite	99.0%	Shouguang Changtai New Materials Co., Ltd.
Barite	95.0%	Tianjin Yandong Haotian Mineral Products Co., Ltd.
Tetrahydrofuran (THF)	99.9%	Merck, Rahway, NJ, USA
Emulsifier-1 (PF-EMUL HT)	/	China Oilfield Services Limited.
Emulsifier-2 (OP-4SA)	/	Jiangsu Hai’an Petrochemical Plant.

a Emulsifier-1 (PF-EMUL HT) and Emulsifier-2 (OP-4SA) are commercial products used for comparison in Section 3.3.1.

**Table 2:** Experimental Instruments

Instrument	Model	Manufacturer
Fourier-transform infrared (FTIR) spectrometer	Nicolet iN10	Thermo Fisher Scientific, Shanghai
Thermogravimetric analyzer(TGA)	TGA-601S	Nanjing Huicheng Instrument and Meter Co., Ltd.
Six-speed viscometer	ZNN-D6	Changzhou Dedu Precision Instrument Co., Ltd., Jiangsu
Gel permeation chromatography	Agilent 1260 Infinity	Agilent Technologies, Inc., Santa Clara, CA, USA
High-temperature roller heating furnace	GRL-BX3	Shandong Meike Instrument Co., Ltd.
Electrical stability tester	FANN 208557	Shenzhen PMZ Technology Inc.
HTHP filtration tester	QD-GGS71-B	Beijing Heng Aode Technology Co., Ltd.
Nanoparticle size analyzer	Litesizer 500	Anton Paar, Shanghai
Spinning Drop Tensiometer	YP-ZL10	Shandong Jiuzhang Scientific Instrument Co., LTD
Adjustable-angle static sag test stand	Custom-built	Custom-built (angle adjustable from 0° to 90°)

## 2.2 Synthesis of PSNA Copolymer

A series of poly­(St-NIPAM-AMPS) copolymers was synthesized via free-radical solution polymerization. The polymerization reaction is shown in Figure 1. Four monomer molar ratios (St:NIPAM:AMPS) were investigated: 1:1.5:1, 1:1:1, 1:2:1, and 1:1.5:0.5. In a typical synthesis, St, NIPAM, and AMPS were dissolved in toluene at a total monomer concentration of 20 wt % and a monomer molar ratio of St:NIPAM:AMPS = 1:1.5:1. The mixture was transferred into a three-necked round-bottom flask equipped with a mechanical stirrer, a reflux condenser, and a nitrogen inlet. After purging with N_2_ for 30 min, the initiator AIBN (0.5 wt % relative to total monomers) was added. The reaction was carried out at 70 °C for 8 h under continuous stirring and an N_2_ atmosphere. After polymerization, the product was precipitated in excess *n*-hexane, filtered, washed repeatedly with *n*-hexane and deionized water, and dried under vacuum at 50 °C for 24 h to afford the copolymer as a white powder, denoted as PSNA. For comparison, a control copolymer without the NIPAM unit, designated PSA (St: AMPS = 1:1), was synthesized following the same procedure as PSNA-2, using 3.34 g of St and 6.66 g of AMPS (total monomer 10 g) in 40.0 g of toluene. The specific feed amounts for each monomer ratio are summarized in Table 3.

**Figure 1:**
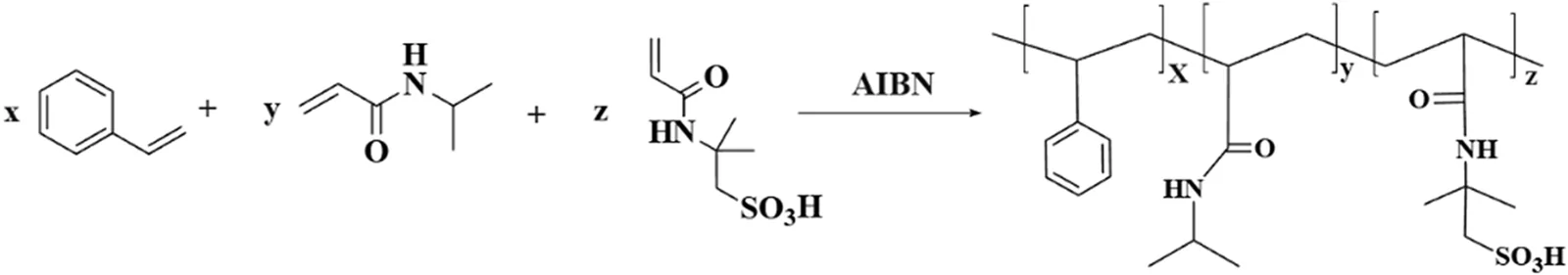
Schematic diagram of the polymerization reaction of PSNA.

**Table 3:** Feed Composition for the Synthesis of PSNA and PSA Copolymers with Different Monomer Molar Ratios (Total Monomer = 10 g, Total Monomer Concentration = 20 wt % in Toluene)

Sample	St:NIPAM:AMPS (mol)	St (g)	NIPAM (g)	AMPS (g)	Toluene (g)
PSNA-1	1:1.5:1	2.16	3.53	4.31	40.0
PSNA-2	1:1:1	2.45	2.67	4.88	40.0
PSNA-3	1:2:1	1.94	4.21	3.85	40.0
PSNA-4	1:1.5:0.5	2.76	4.50	2.74	40.0
PSA	1:0:1	3.34	0	6.66	40.0

## 2.3 Characterization

### 2.3.1 Fourier-Transform Infrared (FTIR) Spectroscopy

The molecular structure of the synthesized PSNA copolymer was characterized by FTIR spectroscopy using a Nicolet iN10 spectrometer. A standard KBr pellet method was employed. Approximately 2 mg of the dried PSNA powder was thoroughly ground with 200 mg of spectroscopic-grade KBr in an agate mortar. The homogeneous mixture was then pressed into a transparent pellet under a hydraulic press at approximately 10 MPa for 2 min. The pellet was placed in the sample holder, and the spectrum was recorded in transmission mode over a wavenumber range of 4000 to 400 cm^–1^ at a resolution of 4 cm^–1^. A total of 32 scans were accumulated and averaged to achieve a high signal-to-noise ratio. A background spectrum was collected using a pure KBr pellet before sample measurement and automatically subtracted from the sample spectrum.

### 2.3.2 Thermogravimetric Analysis (TGA)

Thermal stability was assessed using a TGA-601S thermogravimetric analyzer. About 20 mg of sample was placed in an Al_2_O_3_ crucible. The measurement was performed under an N_2_ atmosphere from room temperature to 650 °C at a heating rate of 10 °C/min. Weight loss curves and their derivatives were recorded.

### 2.3.3 Molecular Weight Determination

Molecular weights and polydispersity indices were determined via gel permeation chromatography (Agilent 1260 Infinity). HPLC-grade THF was used as the mobile phase at a flow rate of 1.0 mL/min. Calibration was carried out with narrow-dispersity polystyrene standards. The PSNA sample was dissolved in THF at ∼2.0 mg/mL and filtered through a 0.45 μm PTFE syringe filter before injection. Although PSNA contains polar AMPS units, the sample dissolved completely in THF after stirring at room temperature for 24 h, as confirmed by visual inspection and unimodal GPC peaks, ensuring reliable molecular weight determination.

## 2.4 Preparation and Formulation of OBDFs

The formulation of the PSNA-2-based OBDF was designed to achieve a balance between emulsion stability, rheological properties, and filtration control at a low OWR. The final optimized formulation (OWR = 70:30, density = 1.8 g/cm^3^) is summarized in Table 4, and the drilling fluid was prepared following a sequential addition procedure to ensure proper dissolution of each component and stepwise formation of a stable invert emulsion. First, white oil (continuous phase) was placed in a high-speed mixing cup, and PSNA-2 (6 wt % relative to the oil phase) was added and stirred at 10,000 rpm for 20 min. Subsequently, organoclay (3.0 vol% of total fluid) was added and stirred for an additional 30 min to build viscosity, followed by the sequential addition of cetyltrimethylammonium bromide (2.0 vol%) and lime (3.5 vol%) under stirring at 8000 rpm for 10 min. The brine phase (30 wt % CaCl_2_ solution, 30 vol%) was then slowly introduced while stirring at 10,000 rpm, and emulsification continued for 45 min to form a stable water-in-oil emulsion. Oxidized asphalt (4.2 vol%) was added as a filtration control agent and stirred at 8000 rpm for 20 min, and finally, Barite was gradually added at 4000–6000 rpm to increase the density to 1.8 g/cm^3^, followed by homogenization at 10,000 rpm for 30 min. For comparison, the same procedure was used to prepare formulations containing commercial emulsifiers (PF-EMUL HT or OP-4SA) at the same concentration (6 wt % of oil phase), as well as a blank formulation without any emulsifier. All formulated OBDFs were hot-rolled at specified temperatures (90, 120, 150, or 180 °C) for 16 h to simulate downhole conditions, then cooled, remixed for 5 min at 8000 rpm, and characterized according to API 13B-2 standards.

**Table 4:** Formulation of the PSNA-2-Based OBDF (OWR = 70:30, Density = 1.8 g/cm^3^)­^a^

Additive	Role	Dosage	Step
White Oil	Continuous phase	Balance	1
PSNA-2	Primary emulsifier	6.0 wt % on oil	2
Lime	pH and alkalinity control	3.5 vol %	2
Organoclay	Viscosifier	3.0 vol %	3
30% CaCl_2_ solution	Dispersed Phase	30 vol %	4
Cetyltrimethylammonium bromide	Wetting agent	2.0 vol %	5
Oxidized asphalt	Filtration control agent	4.2 vol %	6
Barite	Density regulator	To 1.8 g/cm^3^	7

a Wt % = weight percentage relative to oil phase; vol % = volume percentage relative to total fluid volume.

## 2.5 Drilling Fluid Performance Evaluation

All performance evaluations detailed in this section were conducted in triplicate. The results are reported as the mean ± standard deviation to ensure statistical reliability.

### 2.5.1 Rheological Property Measurements

The drilling fluid samples were stirred at high speed (11,000 rpm) for 20–30 min in a slurry cup. Subsequently, the homogenized samples were transferred to the test cup of a ZNN-D6 six-speed rotational viscometer. Rheological parameters were measured sequentially at rotational speeds of 600, 300, 200, 100, 6, and 3 rev/min. Apparent viscosity (AV), plastic viscosity (PV), and yield point (YP) were calculated using the following standard formula^30^ and repeated three times to get the average value.

1 $$\mathrm{AV}=0.5\;\Phi_{600}$$

2 $$\mathrm{PV}=\Phi_{600}-\Phi_{300}$$

3 $$\mathrm{YP}=0.511\left(\Phi_{300}-\mathrm{PV}\right)$$

### 2.5.2 Electrical Stability Test (ES)

Emulsion stability was determined by measuring the dielectric demulsification voltage using an electrical stability tester (FANN 208,557, Shenzhen PMZ Technology Inc.) following API Recommended Practice 13B-2. The test procedure was as follows: a 50 mL homogeneous sample of the drilling fluid was placed into the test cup. The sample was then heated to and maintained at 55 ± 1 °C in a constant-temperature water bath for at least 10 min, with gentle manual stirring immediately before testing to ensure uniformity. A concentric cylindrical electrode assembly was inserted into the sample. The voltage was increased linearly at a constant rate of 150 V/s until a sharp drop in current indicated emulsion breakdown, and the maximum voltage was recorded as the demulsification voltage (ES).^31^ This procedure was repeated three times on fresh samples, and the average value was reported.

### 2.5.3 HTHP FL Test

The HTHP FL method introduced in the literature is adopted for evaluation.^32^ Briefly, different concentrations of the PSNA-2 emulsifier (based on oil phase weight) were added to the OBDFs, and each formulation was stirred at 10,000 rpm for 20 min to ensure homogeneous dispersion. The OBDFs were then hot-rolled for 16 h at target temperatures of 90, 120, 150, and 180 °C to simulate downhole aging. After aging and cooling, the HTHP FL was measured using an HTHP filtration tester at the respective aging temperature under a differential pressure of 3.5 MPa. All tests were performed in triplicate, and the average value is reported.

### 2.5.4 Settlement Stability Test

The static sedimentation method introduced in the literature is usually adopted at the drilling site for evaluation, and the SF is used to quantify the sedimentation stability of the drilling fluid.^33^ That is, test samples were subjected to hot rolling at predetermined temperatures for 16 h, followed by static aging at ambient conditions for 24 h. The densities of the top­(ρ_top_) and bottom­(ρ_bottom_) layers were then measured using a drilling fluid densimeter. The SF was calculated as follows^34^

4 $$SF=\frac{\rho_{bottom}}{\rho_{bottom}+\rho_{top}}$$

An SF value greater than 0.52 indicates significant Barite sedimentation within the drilling fluid system, while an SF value of 0.50 signifies optimal sag resistance with no noticeable settlement. To evaluate the effect of inclination angle, additional tests were conducted at vertical (0°), 45°, and horizontal (90°) orientations.

### 2.5.5 Ecotoxicity Test

The impact of OBDF on the environment was evaluated using biological toxicity tests, and the 50% effective concentration (EC_50_) was determined using the fluorescent bacteria method.^35^ The environmental impact of the OBDF was preliminarily evaluated through biological toxicity tests using the luminescent bacteria method, which is a standard and widely accepted assay in the drilling fluid industry for rapid screening. While multispecies ecotoxicity tests provide a more comprehensive assessment, the luminescent bacteria test offers a reliable and standardized indicator for initial environmental compatibility screening.^36^

## 2.6 Interfacial and Microstructural Characterization

Complementary measurements of interfacial properties and colloidal microstructure were carried out to understand how PSNA stabilizes the emulsion and prevents Barite sag.

### 2.6.1 Interfacial Tension Measurement

The interfacial activity of the PSNA emulsifier was quantified by measuring the equilibrium interfacial tension (IFT) between white oil and the 30 wt % CaCl_2_ aqueous phase. Measurements were conducted using a spinning drop tensiometer (YP-ZL10, Shandong Jiuzhang Scientific Instrument Co., LTD) at 25 °C. The IFT value was recorded for the PSNA-2 system (PSNA-2 concentration = 6 wt % of oil phase) to directly assess its interfacial performance. All measurements were performed in triplicate, and the average value is reported.

### 2.6.2 Emulsion Droplet Size Analysis

The droplet size distribution of the freshly prepared invert emulsions was analyzed by laser diffraction using a Mastersizer 3000 particle size analyzer (Malvern Panalytical). To preserve the native droplet morphology and avoid coalescence or disruption during sampling, each emulsion sample was gently diluted with white oil (the continuous phase) and immediately measured without high-speed shearing or centrifugation. The volume-weighted mean diameter (D50) and the polydispersity index (PDI) were recorded to evaluate emulsion uniformity and stability. Measurements were performed in triplicate for each formulation.

### 2.6.3 Zeta Potential and Aggregate Size of Emulsifier Dispersions

The electrokinetic potential (zeta potential) of emulsifier aggregates in the continuous oil phase was measured to investigate the interfacial charge and thermally induced changes. Samples were diluted 100-fold with white oil, ultrasonicated for 30 min to ensure uniform dispersion, and then centrifuged at 3000 rpm for 5 min to remove large particulates (e.g., Barite, organoclay). The zeta potential of the supernatant was determined using a Litesizer 500 nanoparticle size potentiometer (Anton Paar, Shanghai). All measurements were conducted at 25 °C, and each reported value is the average of three independent runs.

### 2.6.4 LCST Measurement

The thermoresponsive phase transition behavior of the PSNA-2 copolymer was evaluated using a Litesizer 500 nanoparticle analyzer equipped with a temperature-controlled cuvette holder and a dedicated transmittance detection module at 500 nm. A 1.0 mg/mL solution of PSNA-2 was prepared in a 30 wt % CaCl_2_ aqueous solution (identical to the dispersed phase used in OBDFs). The solution was transferred into a disposable cuvette and heated from 25 to 75 °C at a ramp rate of 1 °C/min. The LCST was defined as the temperature at which the transmittance decreased to 50% of its initial value.

## 3 Results and Discussion

### 3.1 Composition Optimization

#### 3.1.1 Emulsifier Screening and Selection

The molar ratio of St, NIPAM, and AMPS determines the amphiphilic balance, thermal responsiveness, and interfacial activity of the PSNA copolymer, which directly affects its emulsifying performance in OBDFs. To identify the optimal monomer molar ratio, four PSNA samples (PSNA-1 to PSNA-4) were formulated into OBDFs under fixed benchmark conditions: OWR = 70:30, emulsifier concentration = 6 wt % of oil phase, brine = 30 wt % CaCl_2_ solution, density = 1.8 g/cm^3^ (Barite-weighted). Each formulation was hot-rolled at 180 °C for 16 h. After aging, the emulsion stability, filtration loss, and sag resistance were evaluated following the procedures in Section 2.5 The results are summarized in Table 5.

**Table 5:** Performance of Different PSNA Copolymers (Benchmark Conditions)

Sample	St:NIPAM:AMPS (mol)	ES/V	HTHP FL/mL	SF
PSNA-1	1:1.5:1	715 ± 12	4.8 ± 0.3	0.521 ± 0.005
PSNA-2	1:1:1	879 ± 15	3.2 ± 0.2	0.513 ± 0.003
PSNA-3	1:2:1	822 ± 13	3.6 ± 0.2	0.516 ± 0.004
PSNA-4	1:1.5:0.5	548 ± 9	6.5 ± 0.4	0.531 ± 0.006

As shown in Table 5, the demulsification voltage varied significantly with monomer composition. PSNA-2 (St:NIPAM:AMPS = 1:1:1) exhibited the highest ES of 879 V, along with the lowest HTHP FL of 3.2 mL and an SF of 0.513, meeting all industry acceptance criteria; therefore, PSNA-2 was selected as the optimal copolymer. Interestingly, the demulsification voltage did not increase monotonically with NIPAM content: PSNA-2 (1:1:1) gave the highest ES, followed by PSNA-3 (1:2:1, 822 V), while PSNA-1 (1:1.5:1) exhibited a lower value (715 V). The nonmonotonic trend of ES with NIPAM content can be rationalized as follows: at a moderate NIPAM ratio (PSNA-1, 1:1.5:1), the polymer chains suffer from insufficient hydrophobic association upon thermal collapse due to a suboptimal hydration/dehydration balance, leading to interfacial defects. When NIPAM is increased to 1:2:1 (PSNA-3), the collective dehydration and hydrophobic driving force overcompensate for the initial defects, yielding a denser film than PSNA-1. The optimal balance is achieved at 1:1:1 (PSNA-2), where sufficient thermal responsiveness is provided without excessive chain aggregation that might disrupt uniform interfacial packing.

#### 3.1.2 Determination of Optimal Emulsifier Dosage

After PSNA-2 was identified as the optimal copolymer composition, its concentration in the oil phase was optimized. The benchmark conditions used in Section 3.1.1 were maintained: OWR = 70:30, brine = 30 wt % CaCl_2_ solution, density = 1.8 g/cm^3^, aging at 180 °C for 16 h. PSNA-2 concentration was varied from 2 to 10 wt % of the oil phase. The performance metrics, including ES, HTHP FL, SF, and yield point (YP), are summarized in Table 6.

**Table 6:** Effect of PSNA-2 Concentration­(wt% of Oil) on OBDF Performance (OWR = 70:30, Aged at 180 °C for 16 h)

PSNA-2/wt %	AV/mPa·s	PV/mPa·s	YP/Pa	HTHP FL/mL	ES/V	SF
2	18.5	15.3	3.2	8.2	412	0.533
4	27.2	21.4	5.8	5.1	673	0.524
6	34.6	26.5	8.1	3.2	879	0.513
8	38.9	29.7	9.2	3.0	855	0.511
10	43.5	33.1	10.5	3.1	841	0.512

At a low concentration of 2 wt %, the ES was only 412 V, far below the industry requirement of 800 V, and the SF exceeded 0.52, indicating severe Barite sag. This is attributed to insufficient interfacial coverage and weak emulsion film strength. Increasing the concentration to 4 wt % improved ES to 673 V and reduced HTHP FL to 5.1 mL, but the SF remained marginally above 0.52. At 6 wt %, all three mandatory criteria were met: ES reached 879 V (>800 V), HTHP FL decreased to 3.2 mL, and SF dropped to 0.513 (<0.52). Further increasing the concentration to 8 and 10 wt % yielded only marginal improvements in ES and filtration control, but caused a notable increase in rheological parameters—AV rose from 34.6 to 43.5 mPa·s, PV from 26.5 to 33.0 mPa·s, and YP from 8.1 to 10.5 Pa. Such excessive rheology could lead to higher pumping pressure and increased equivalent circulating density (ECD) in field applications. Therefore, 6 wt % was selected as the optimal PSNA-2 concentration for subsequent OWR optimization.

#### 3.1.3 Optimization of Oil-to-Water Ratio

With the optimal PSNA-2 concentration fixed at 6 wt % of the oil phase, the OWR was varied from 90:10 to 50:50 to determine the minimum feasible OWR for the PSNA-2-based OBDF system. The other benchmark conditions remained unchanged: brine = 30 wt % CaCl_2_ solution, density = 1.8 g/cm^3^, aging at 180 °C for 16 h. The performance metrics are summarized in Table 7.

**Table 7:** Optimization of the OWR

OWR	AV/mPa·s	PV/mPa·s	YP/Pa	HTHP FL/mL	ES/V	SF
90:10	28.3	23.1	5.2	2.4	1120	0.525
80:20	31.5	24.7	6.8	2.8	968	0.519
70:30	34.6	26.5	8.1	3.2	879	0.513
60:40	39.8	29.5	10.3	4.7	715	0.522
50:50	46.2	33.6	12.6	7.2	553	0.534

As the water fraction increased, ES decreased monotonically because the larger interfacial area required stabilization by a fixed amount of emulsifier. OWRs of 90:10 and 80:20 gave ES values well above 800 V (1120 and 968 V) with low filtration losses, indicating robust stability.^37^ At OWR = 70:30, the ES remained acceptable (879 V), HTHP FL was 3.2 mL, and SF was 0.513, meeting all industry criteria. However, further reducing the OWR to 60:40 caused ES to fall below 800 V (715 V) and SF to rise to 0.521, signaling a risk of Barite sag; at 50:50, the performance deteriorated further (ES = 553 V, SF = 0.534, HTHP FL = 7.2 mL), making the formulation unsuitable for field use. Rheologically, AV, PV, and YP increased steadily with water content, but at 60:40 and 50:50, the YP exceeded 10 Pa, potentially leading to excessive pumping pressure in deep wells.

Based on these results, OWR = 70:30 was identified as the minimum feasible oil-to-water ratio for the PSNA-2 stabilized OBDF system. This low OWR offers significant economic and environmental benefits by reducing base oil consumption while maintaining emulsion stability, filtration control, and sag resistance at 180 °C.

### 3.2 Characterization

#### 3.2.1 FTIR Analysis of PSNA

Figure 2 shows the FTIR spectrum confirming the molecular structure of the synthesized PSNA. The spectrum exhibits a broad absorption band at 3429 cm^–1^ attributable to the N–H stretching vibration of the amide groups from NIPAM and AMPS units. The characteristic absorptions of the styrene component are clearly identified by the weak aromatic = C–H stretching at 3058 cm^–1^ and the strong monosubstituted benzene ring out-of-plane bending vibration at 699 cm^–1^, along with the C=C skeletal vibration at 1452 cm^–1^. The strong bands at 2928 and 2853 cm^–1^ correspond to the asymmetric and symmetric C–H stretching of the saturated aliphatic backbone, respectively. Crucially, the successful incorporation of the thermoresponsive NIPAM unit is evidenced by the amide I and II bands at 1663 and 1548 cm^–1^, respectively, and most notably by the characteristic isopropyl splitting doublet at 1385 and 1365 cm^–1^ attributed to the symmetric bending of the −CH­(CH_3_)_2_ group. Furthermore, the presence of the ionic sulfonate groups from AMPS is confirmed by the strong asymmetric and symmetric S=O stretching vibrations at 1179 and 1038 cm^–1^, respectively. All these characteristic peaks collectively confirm the successful ternary copolymerization of St, NIPAM, and AMPS into the PSNA structure.

**Figure 2:**
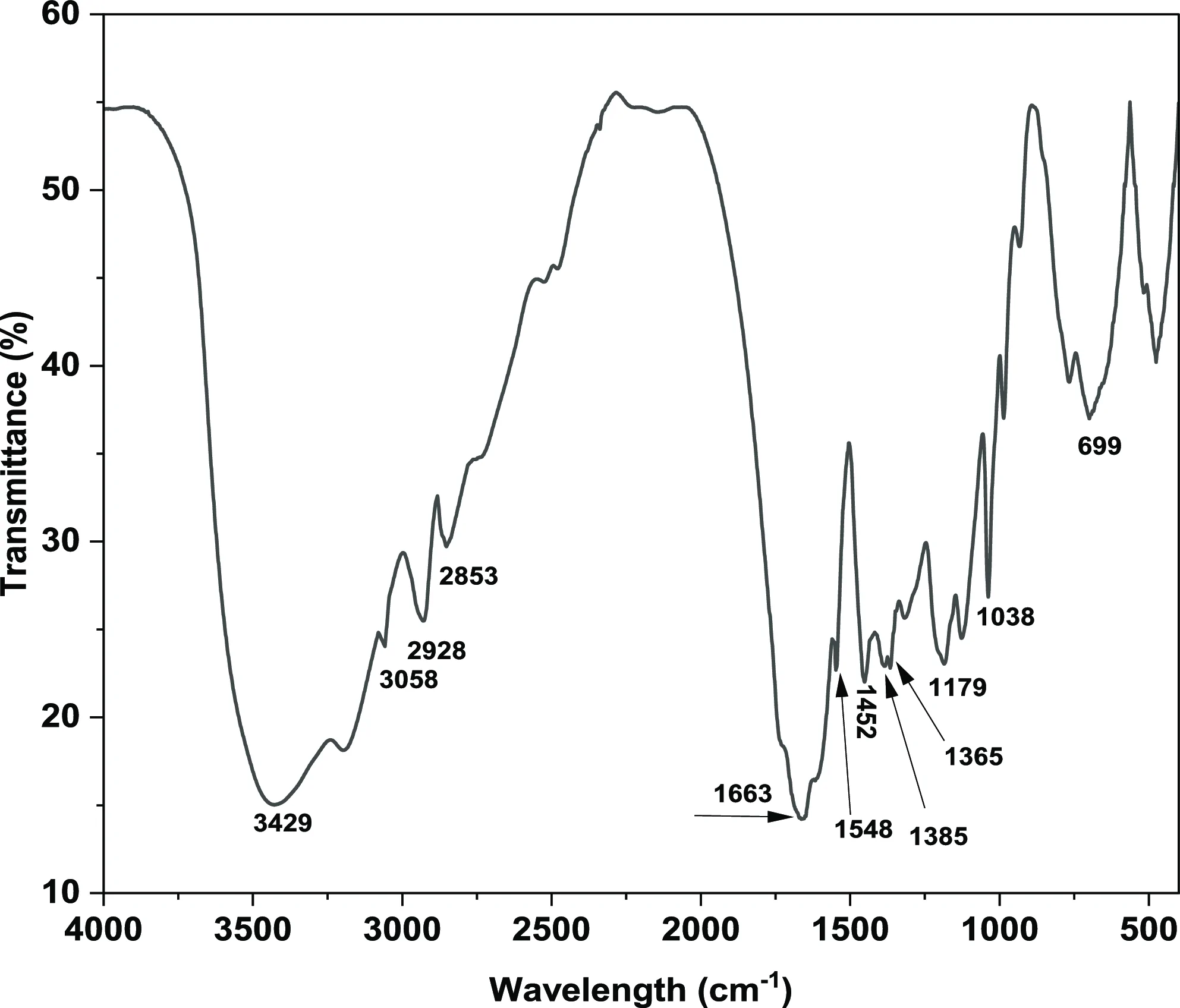
FTIR spectrum of PSNA.

#### 3.2.2 ^1^H NMR analysis of PSNA

As shown in Figure 3, the aromatic protons of the styrene units give two distinct broad singlets at 6.812 and 6.681 ppm, corresponding to the o-/p-protons and m-protons on the benzene ring, respectively. The AMPS component is identified by a weak, broad signal at 3.049 ppm (assigned to −CH_2_–SO_3_H) and a strong, sharp singlet at 1.526 ppm (assigned to −C­(CH_3_)_2_−). The signal at 1.526 ppm exhibits the highest peak intensity in the entire spectrum, consistent with the six methyl protons of the AMPS unit. The NIPAM isopropyl group gives two methyl doublets at 1.156 and 1.061 ppm, and a broad methine signal at 3.903 ppm. No signals are observed above 8.0 ppm, indicating the absence of aldehydes or other high-shift impurities. All characteristic peaks confirm the successful incorporation of the three monomers into the PSNA copolymer.

**Figure 3:**
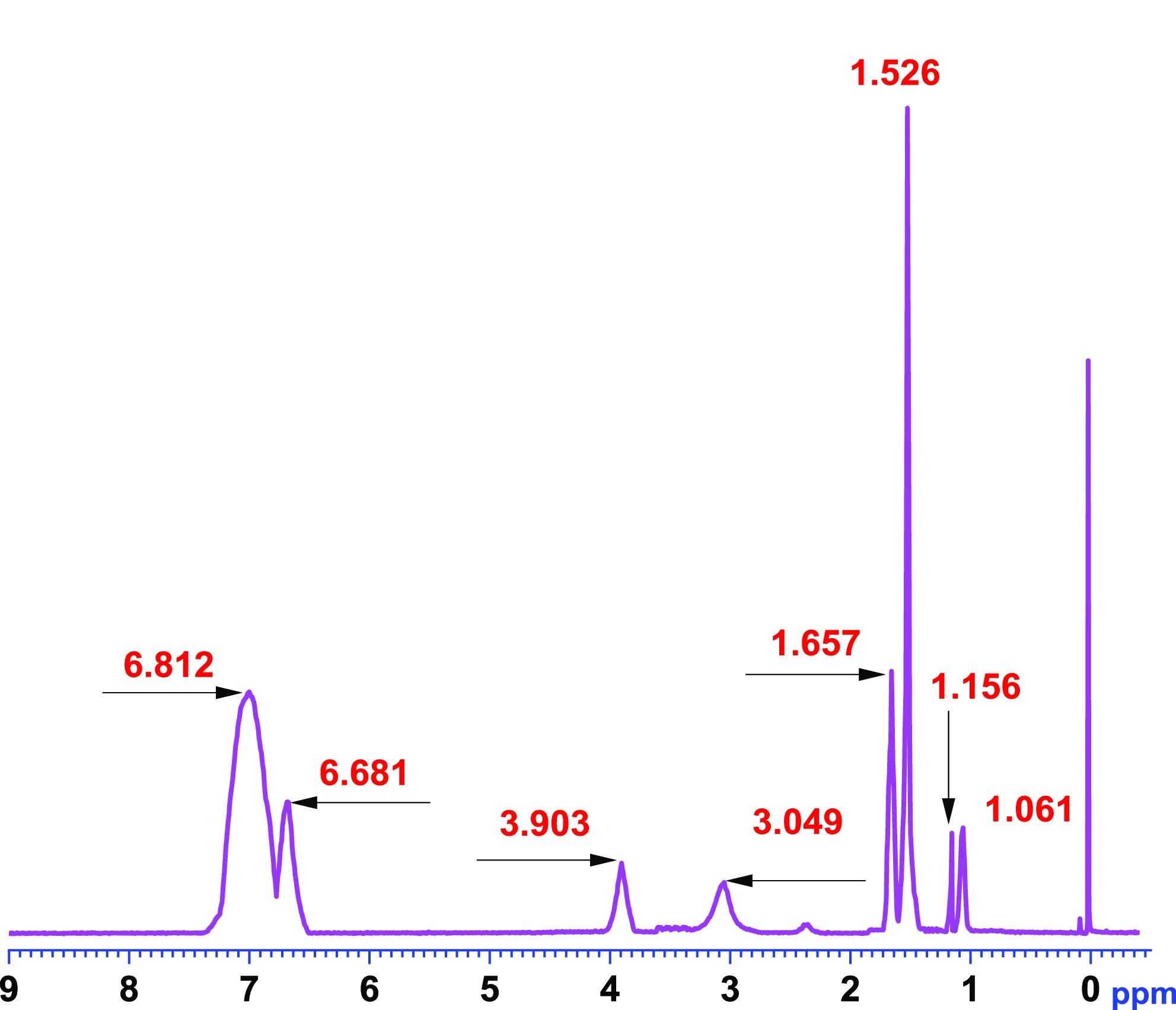
^1^H NMR spectrum of PSNA.

#### 3.2.3 TGA

Figure 4 shows the TGA and DTG curves of PSNA-2, which indicate that the degradation proceeds in three distinct stages. In the first stage, before 140 °C, a mass loss of 4.41% occurs due to evaporation of residual solvents and absorbed moisture.^38^ The second stage, between 140 and 520 °C, is the major decomposition region, where the DTG curve shows the maximum mass loss rate at 260 °C, attributed to the cleavage of labile side chains (amide groups of NIPAM and sulfonate groups of AMPS). At 520 °C, the residual mass is 22.08%. In the third stage, after 520 °C, the mass loss continues gradually to 16.02% at 650 °C, corresponding to carbonization and further decomposition of residual char; the final char residue is approximately 16.0%. Importantly, the mass retention at 180 °C (the maximum aging temperature in this study) exceeds 90%, confirming that PSNA-2 can remain thermally stable and functional under the target downhole conditions (180–220 °C).

**Figure 4:**
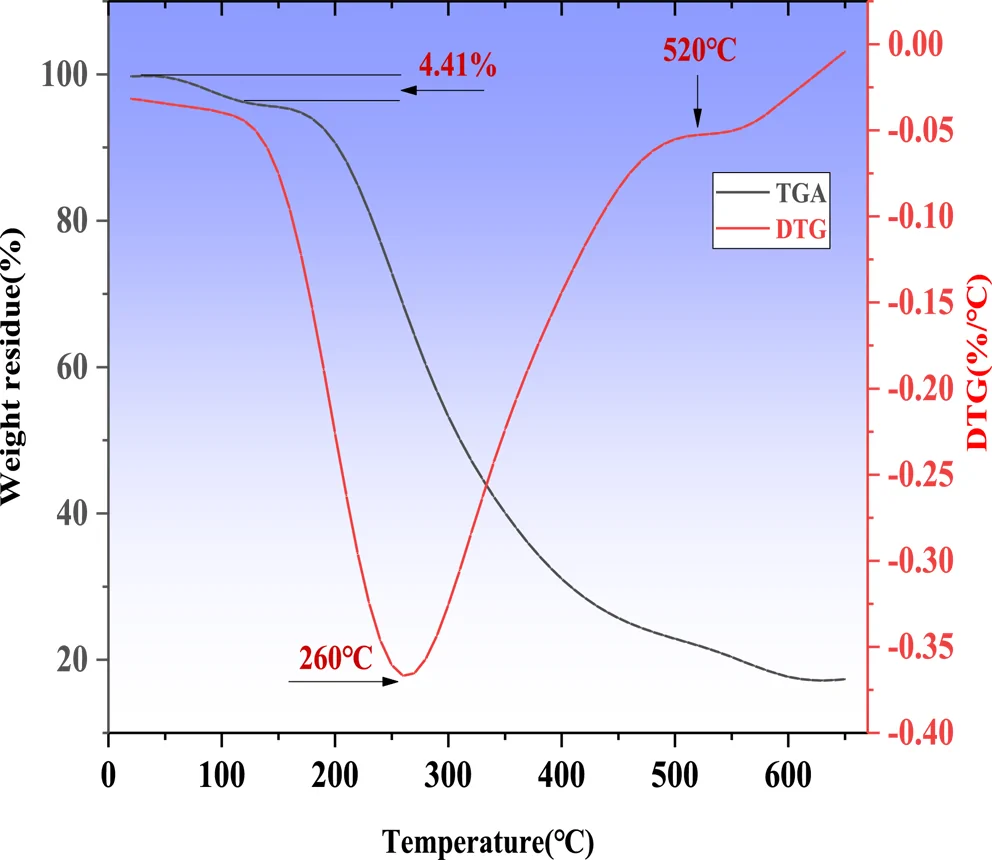
TGA curve of PSNA.

#### 3.2.4 GPC

The molecular weights and PDI of the synthesized PSNA copolymers and the control P­(St-AMPS) were determined by GPC, and the results are summarized in Table 8. Table 8 revealed that all PSNA copolymers had *M_n_* values of 2.59–3.45 × 10^4^ g/mol and PDIs of 2.13–2.22. These values are typical for conventional free-radical solution polymerization carried out in toluene at a monomer concentration of 20 wt % and an AIBN initiator loading of 0.5 wt %. The moderate PDI (around 2.1–2.2) indicates that the polymerization proceeded under steady, controlled conditions without significant chain transfer to solvent or premature termination, which is consistent with the mild reaction temperature (70 °C) and the nonliving radical mechanism. PSNA-2 showed an *M_n_* of 3.32 × 10^4^ g/mol and a PDI of 2.14. The control P­(St-AMPS) exhibited nearly identical molecular parameters (*M_n_* = 3.21 × 10^4^ g/mol, PDI = 2.14), confirming that the emulsification performance differences are not due to chain length effects. PSNA-4 exhibits a slightly higher *M_n_* than others, which is attributed to its lower AMPS content (0.5 molar ratio); AMPS is known to participate in chain transfer reactions during radical polymerization, so reducing its fraction decreases transfer events and allows higher molecular weight buildup.

**Table 8:** Molecular Weights and Polydispersity Index (PDI) of the Synthesized PSNA Copolymers and the Control P­(St-AMPS)

Sample	St:NIPAM:AMPS (mol)	*M_n_* (g/mol)	*M_w_* (g/mol)	PDI
PSNA-1	1:1.5:1	27,894	61,088	2.19
PSNA-2	1:1:1	33,215	71,080	2.14
PSNA-3	1:2:1	26,498	58,825	2.22
PSNA-4	1:1.5:0.5	35,841	76,341	2.13
PSA	1:0:1	32,451	68,751	2.14

### 3.3 Performance Evaluation of OBDFs

The overall performance of the PSNA-2-based OBDF was systematically evaluated, including thermal stability over a range of aging temperatures, comparison with a commercial high-temperature emulsifier, sag stability under inclined conditions, and environmental toxicity.

#### 3.3.1 Thermal Stability and Aging Temperature Tolerance

The thermal stability of the PSNA-2 based OBDF was evaluated by dynamic aging at 180 °C for 16 h, and its performance was compared with a base formulation (without any emulsifier), a control copolymer without the thermoresponsive NIPAM unit, PSA, synthesized as described in Section 2.2, and two commercial high-temperature emulsifiers: PF-EMUL HT (a surfactant modified by plant oil fatty acids) and OP-4SA (an ammonium salt of polyethylene ether sulfate). The results are summarized in Table 9.

**Table 9:** Comprehensive Performance Comparison of Different OBDF Systems after Hot-Rolling at 180 °C for 16 h

System	AV (mPa·s)	PV (mPa·s)	YP (Pa)	ES (V)	HTHP FL (mL)	SF
Blank (no emulsifier)	25.5	21.4	4.1	496	5.2	0.533
PSA	26.8	22.3	4.5	554	5.8	0.529
PF-EMUL HT	33.5	26.3	7.2	849	3.6	0.515
OP-4SA	31.5	25.6	5.9	815	3.8	0.517
PSNA-2	37.0	27.9	9.1	879	3.2	0.512

As shown in Table 9, the base formulation (no emulsifier) failed all criteria (ES = 496 V, HTHP FL = 5.2 mL, SF = 0.533). Interestingly, the PSA-stabilized system exhibited even higher HTHP FL (5.8 mL) than the blank mud (5.2 mL). This is because PSA, lacking the thermoresponsive NIPAM unit, acts as a weak amphiphilic destabilizer at high temperature: it adsorbs at the oil–water interface but cannot form a compact film, thereby disrupting the natural interfacial packing of other components (e.g., organoclay and asphalt) and exacerbating filtration loss. Both commercial emulsifiers met the minimum criteria, with PF-EMUL HT (ES = 849 V, FL = 3.6 mL, SF = 0.515) outperforming OP-4SA. Notably, the PSNA-2 formulation gave the best overall performance under the conditions tested: ES = 879 V, HTHP FL = 3.2 mL, SF = 0.512, and acceptable rheology (AV = 37.0 mPa·s, YP = 9.1 Pa). While acknowledging that the commercial emulsifiers were used as received with unknown active ingredient contents, this comparison based on equal mass loading demonstrates that PSNA-2 is at least comparable to, and in several metrics superior to, the benchmark commercial products. Thus, PSNA-2 exhibits excellent thermal stability at 180 °C, which we attribute to its thermoresponsive adaptive interfacial reinforcement—a feature completely absent in the PSA control.

It should be noted that the commercial emulsifiers were used as received, and their active ingredient contents may be lower than 100%. The comparison presented here is therefore a practical engineering benchmark based on equal mass loading of the oil phase, rather than an equal-active-matter basis.

#### 3.3.2 Sag Stability under Elevated Temperatures and Inclined Conditions

Barite sag was evaluated using the SF (SF > 0.52 indicates significant sag) under varying aging temperatures (90–180 °C) and inclination angles (0–90°) after hot-rolling at 180 °C. All samples were first hot-rolled for 16 h at the designated temperature, then statically aged for 24 h at room temperature before SF measurement. The results are shown in Figures 5a-b, respectively.

**Figure 5:**
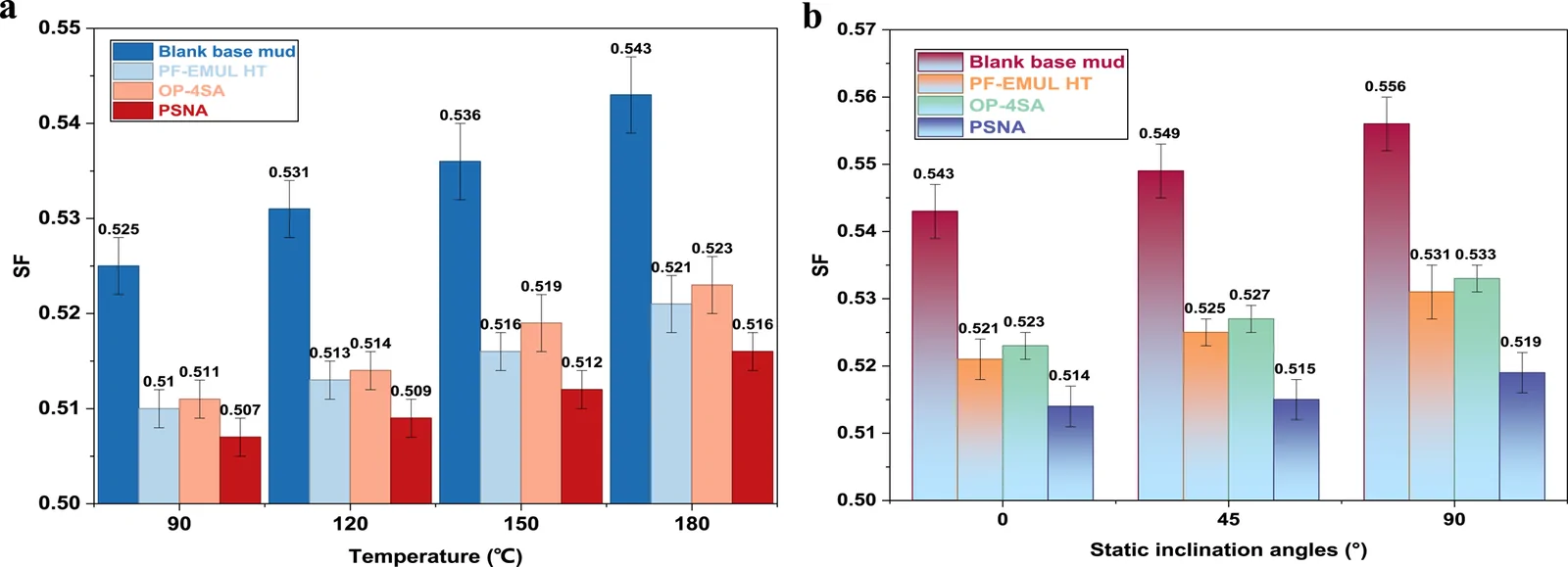
(a) Variation of SF with thermal aging temperature for different OBDF systems. (b) SF of different OBDF systems under various static inclination angles after hot-rolling at 180 °C.

For all systems, SF increased with temperature due to reduced oil viscosity and weakened emulsion network, and also increased with inclination angle as gravity-induced settling became more severe. The blank base mud (no emulsifier) exceeded SF > 0.52 already above 120 °C and failed at all angles (SF > 0.54). Commercial emulsifiers PF-EMUL HT and OP-4SA marginally met the criterion at 150 °C (SF = 0.516 and 0.519, respectively) but approached or exceeded 0.52 at 180 °C; they performed acceptably at vertical orientation but exceeded SF = 0.52 at 45° and 90°. In contrast, the PSNA-2-based OBDF maintained SF ≤ 0.514 across the entire temperature range, with the smallest increment from 0.507 (90 °C) to 0.516 (180 °C), and remained below the 0.52 threshold at all angles (SF = 0.514 at 0°, 0.515 at 45°, and 0.519 at 90°). This superior performance is attributed to the synergistic contributions of the three monomers: St provides strong hydrophobic anchoring to prevent emulsifier desorption; NIPAM undergoes thermoresponsive collapse that densifies the interfacial film upon heating; and AMPS offers electrostatic repulsion and steric stabilization, collectively maintaining Barite suspension even under high-temperature and deviated conditions.

#### 3.3.3 Environmental Toxicity Assessment

According to the industry standard Q/SY 111–2007 (Luminescent bacteria test), the EC_50_ values of the drilling fluids were determined.^39^ The luminescent bacteria method has been widely adopted for rapid environmental screening of drilling fluids and related oilfield chemicals,^21^ and the results are listed in Table 10. The EC_50_ results of the three groups of samples are as follows: 24,800 mg/L­(Blank), 34,520 mg/L­(PF-EMUL HT), 33,890 mg/L­(OP-4SA), and 36,860 mg/L­(PSNA-Optimized Formulation). According to the biological toxicity grade classification standard, when the EC_50_ was larger than 25,000 mg/L, OBDF was nontoxic. If the EC_50_ exceeded 30,000 mg/L, the emission standard was achieved.^40^

**Table 10:** Biotoxicity of Different Drilling Fluid Samples

Sample	EC_50_, mg/L	Biological toxicity
Blank (no emulsifier)	24,800	No toxicity
PF-EMUL HT	34,520	No toxicity
OP-4SA	33,890	No toxicity
PSNA-2	36,860	No toxicity

### 3.4 Emulsion Stabilization Mechanism

To elucidate the underlying mechanisms responsible for the exceptional performance of the PSNA-based OBDF—particularly its thermal stability, low filtration loss, and sag resistance—a series of complementary analyses was conducted. These include the LCST of the copolymer, interfacial tension reduction, droplet size evolution, and zeta potential measurements. Together, these investigations reveal a thermally triggered interfacial self-reinforcement mechanism, wherein the collapse of NIPAM segments above the LCST drives densification of the emulsifier film at elevated temperatures, thereby counteracting the increased interfacial stress associated with low OWR.

#### 3.4.1 Thermoresponsive Conformational Transition of PSNA

As shown in Figure 6, the LCST of PSNA-2 in 30 wt % CaCl_2_ brine is determined to be 54 °C, markedly higher than that of pure PNIPAM (∼32 °C in pure water). This elevation arises from the strong hydration of AMPS sulfonate groups, which raises the dehydration energy barrier of NIPAM segments. Meanwhile, the hydrophobic St units partially offset this increase by enhancing chain aggregation. The net LCST of 54 °C thus reflects a balanced antagonism between the hydrophilic AMPS and hydrophobic St comonomers. Although the CaCl_2_ brine exerts a salting-out effect that typically depresses LCST, the AMPS-dominated hydration effectively counteracts this depression. Strategically, this LCST is well above ambient temperature for facile surface emulsification, yet far below downhole conditions (180 °C), ensuring complete chain collapse and dense interfacial packing at elevated temperature.

**Figure 6:**
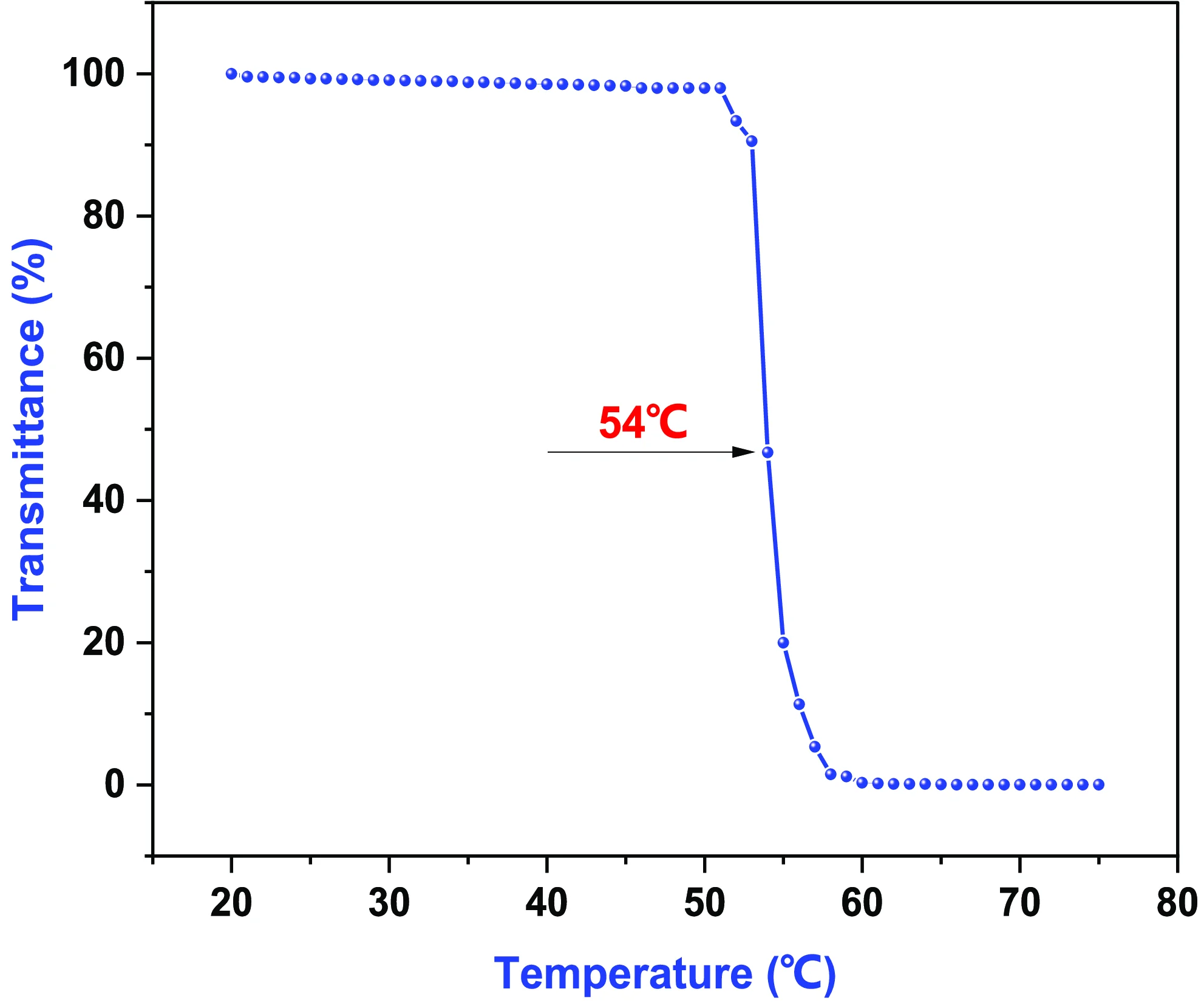
Transmittance at 500 nm as a function of temperature for a 1.0 mg/mL PSNA-2 solution in 30 wt % CaCl_2_ brine.

#### 3.4.2 Interfacial Activity and Droplet Size Evolution

Figure 7a compares the IFT of different emulsifier systems before and after thermal aging (180 °C, 16 h). The blank system (no emulsifier) gave an IFT of 22.41 mN/m but completely demulsified after aging, so its aged IFT could not be measured. Notably, the IFT reduction of PSNA-2 from 3.88 mN/m (fresh) to 2.65 mN/m (after aging at 180 °C) cannot be simply attributed to thermal annealing. The control PSA, lacking the NIPAM unit, showed an opposite trend (IFT increased from 6.26 to 7.58 mN/m under identical conditions), indicating that the NIPAM-mediated coil-to-globule transition is the key driver for interfacial densification. This conclusion is further supported by the LCST measurement (Figure 6), which confirms that at 180 °C, PSNA-2 exists entirely in its collapsed globular state, maximizing hydrophobic interactions at the interface.

**Figure 7:**
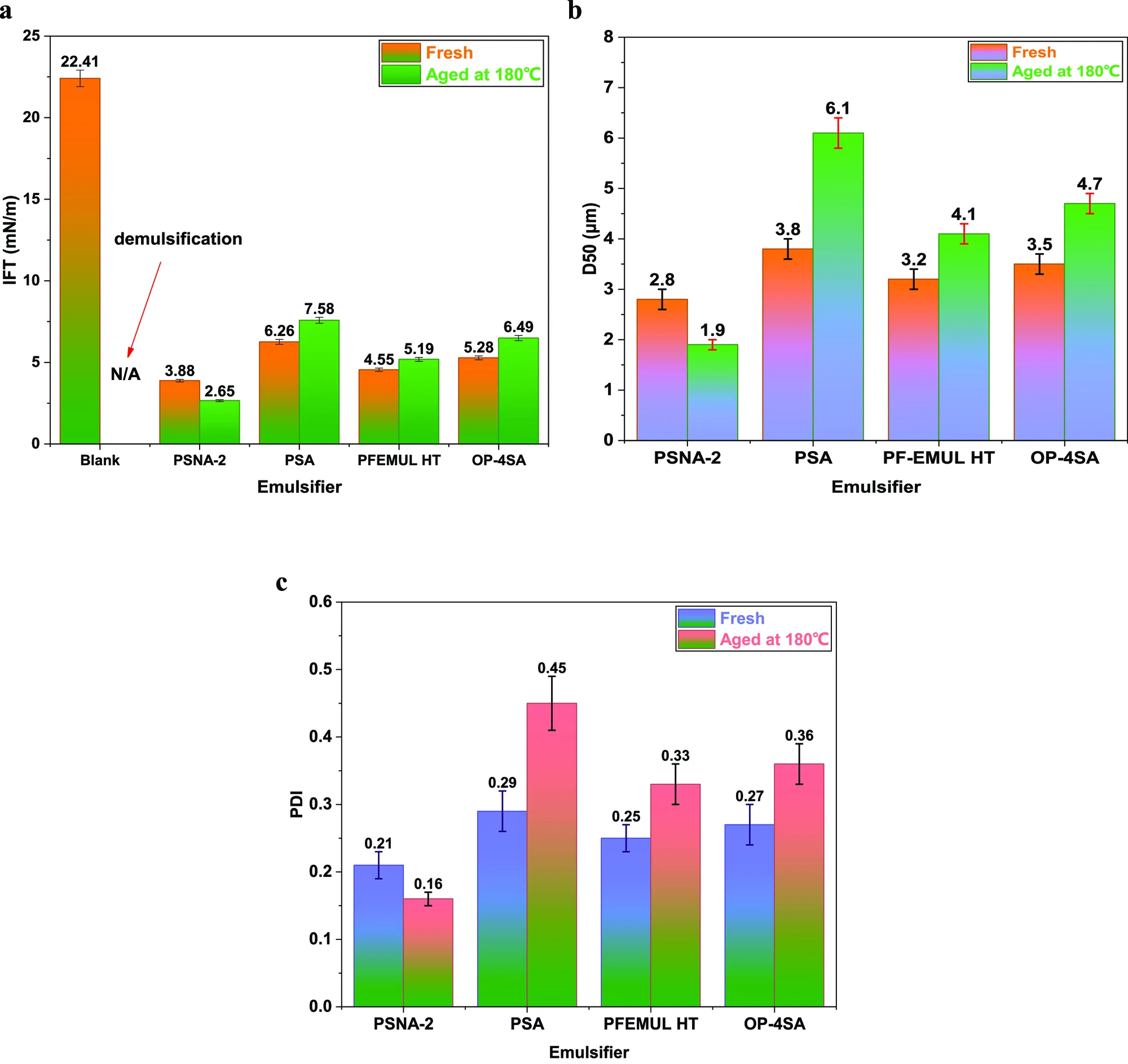
(a) IFT of different emulsifier systems before and after thermal aging at 180 °C for 16 h. (b) D50 of emulsion droplets before and after thermal aging at 180 °C for 16 h. (c) PDI of emulsion droplets before and after thermal aging at 180 °C for 16 h.

Figure 7b,c present the D50 and PDI for each stable emulsion system. The blank system is omitted because it failed to form a stable emulsion. For PSNA-2, D50 decreased from 2.80 μm (fresh) to 1.90 μm (aged), and PDI narrowed from 0.21 to 0.16, reflecting a finer and more uniform droplet population after thermal treatment. Conversely, PSA-stabilized droplets coarsened significantly (D50:3.80 → 6.10 μm; PDI: 0.29→0.45). Both commercial emulsifiers also showed coarsening: PF-EMUL HT (D50:3.20 → 4.10 μm; PDI: 0.25→0.33) and OP-4SA (D50:3.50 → 4.70 μm; PDI: 0.27 → 0.36). These results confirm that the thermoresponsive collapse of NIPAM in PSNA-2 not only lowers IFT further but also densifies the interfacial film, producing smaller, more uniform droplets with enhanced thermal stability.

#### 3.4.3 Enhanced Electrostatic Stabilization Upon Thermal Collapse

The strongly ionizable sulfonate groups of AMPS impart a negative charge to the emulsion droplets. Zeta potential measurements of the PSNA-2 stabilized emulsion diluted in the continuous oil phase (white oil) gave an apparent zeta potential of −32.5 mV at 25 °C. After thermal aging at 180 °C, the absolute value increased to −39.4 mV. This increase indicates that the interfacial film becomes more compact and the surface charge density rises, likely because the collapse of NIPAM chains brings more AMPS groups into closer proximity at the interface. Previous studies on colloidal stabilization in nonaqueous systems have established that enhanced electrostatic repulsion, even in low-conductivity media, can provide a significant barrier against droplet coalescence.^41^ This electrosteric stabilization mechanism is uniquely enabled by the ternary copolymer structure and is absent in the PSA control, further corroborating that the NIPAM-mediated thermal collapse is essential for the enhanced electrostatic repulsion observed at elevated temperatures. The enhanced electrostatic repulsion between droplets provides an additional barrier against coalescence, complementing the steric stabilization from the dense polymer film. Zeta potential measurements in the oil phase were performed using a dedicated nonaqueous electrophoresis accessory, which ensures reliable electrokinetic characterization in low-conductivity organic media.

It should be emphasized that the zeta potential values reported herein in the nonpolar oil phase should be interpreted as apparent zeta potentials rather than absolute electrokinetic potentials, given the extremely low dielectric constant and ionic conductivity of the continuous phase. Nevertheless, the observed increase in the absolute value from −32.5 mV to −39.4 mV upon thermal aging is considered a reliable relative indicator of the increased surface charge density at the oil–water interface, which arises from the thermally induced compaction of the PSNA-2 film and the concomitant closer packing of AMPS sulfonate groups. This relative trend is consistent with the concurrent decreases in interfacial tension and droplet size (Figure 7), collectively supporting the proposed thermally triggered self-reinforcement mechanism.

#### 3.4.4 Thermally Triggered Self-Reinforcement

Collectively, these observations support a clear mechanism: at downhole temperature, the collapse of NIPAM chains forces PSNA molecules to reorganize into a more compact interfacial film. This thermally triggered self-reinforcement counteracts the increased interfacial stress associated with low OWR (70:30), maintaining high demulsification voltage, low filtration loss, and sag resistance after prolonged thermal aging. The proposed mechanism is illustrated in Figure 8.

**Figure 8:**
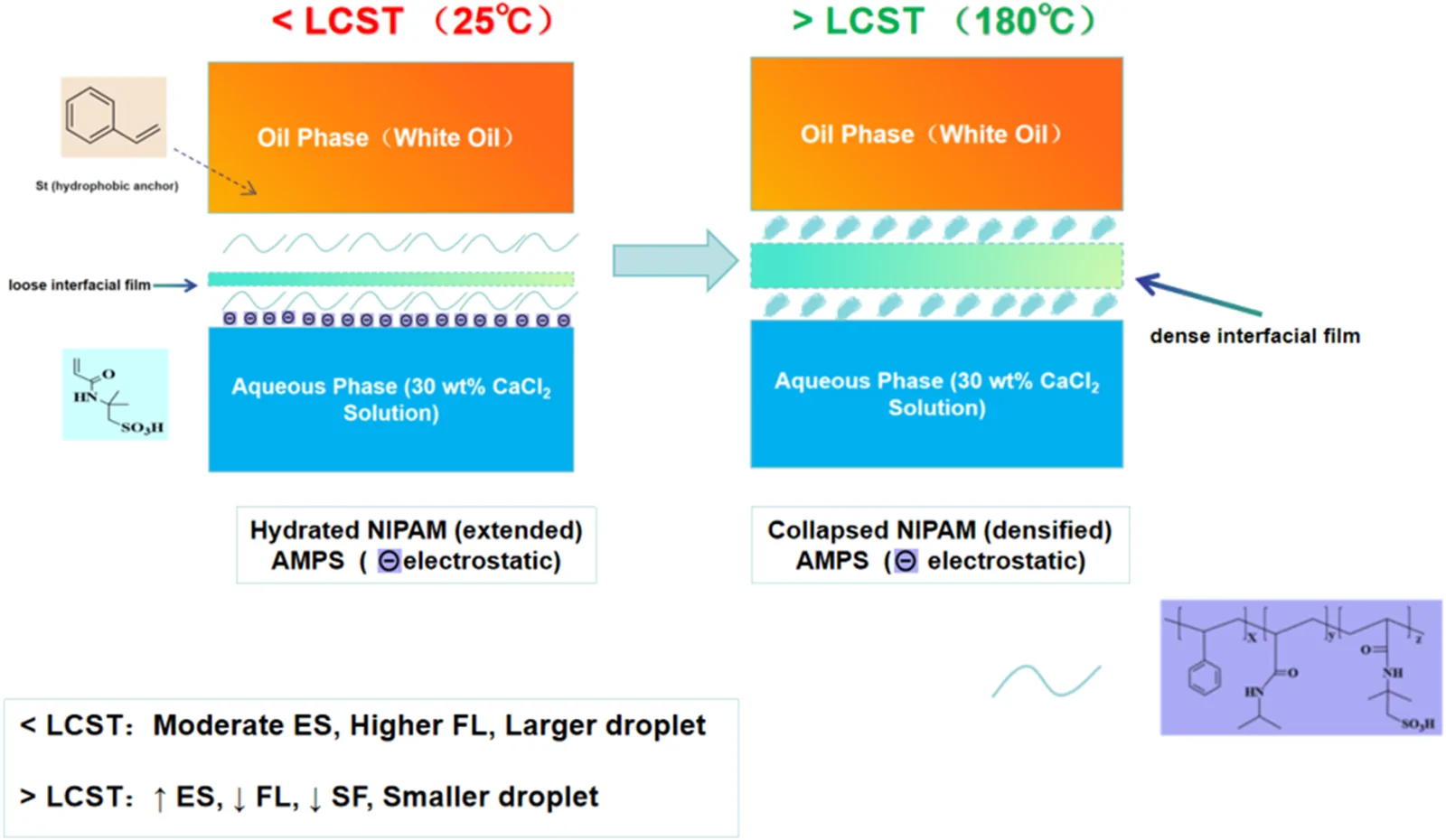
Mechanism of PSNA as a thermoresponsive amphiphilic emulsifier for low-OWR OBDFs.

In contrast to conventional emulsifiers that undergo thermal degradation or desorption, PSNA-2 exploits heat as an activating stimulus. The LCST of 54 °C ensures that at ambient temperature, the polymer remains hydrated and extended, facilitating initial emulsification with low viscosity. Upon entering the high-temperature formation, the NIPAM segments collapse, driving the polymer chains to pack densely at the oil–water interface. This thermally triggered transition effectively counteracts the increased interfacial stress associated with the low OWR of 70:30, as evidenced by the maintenance of high ES (879 V) and low HTHP FL (3.2 mL) even after 16 h at 180 °C.

## 4 Conclusions and Recommendations

A thermoresponsive amphiphilic copolymer, poly­(St-NIPAM-AMPS) (PSNA), was successfully synthesized via a straightforward free-radical solution polymerization of St, NIPAM, and AMPS. Through systematic optimization of the monomer molar ratio, emulsifier concentration, and OWR, the optimal formulation—PSNA-2 at a dosage of 6 wt % relative to the oil phase and an OWR of 70:30—was identified. The PSNA-2-based OBDF exhibited outstanding performance after dynamic aging at 180 °C for 16 h, achieving an ES of 879 V, an HTHP FL of 3.2 mL, and an SF of 0.513. This performance, achieved at a reduced OWR with a relatively low emulsifier loading, represents a significant advancement over conventional emulsifier systems that typically require higher OWRs (80:20 to 90:10) or more complex formulations.

Mechanistically, comprehensive characterization—including FTIR, ^1^H NMR, TGA, interfacial tension, droplet size analysis, zeta potential measurements, and LCST determination—revealed a thermally triggered interfacial self-reinforcement mechanism. The LCST of PSNA-2 was determined to be 54 °C, strategically positioned between ambient temperature (facilitating initial emulsification with extended polymer chains) and downhole conditions (≥150 °C, where the NIPAM segments collapse). Above the LCST, the dehydration and collapse of NIPAM chains drive the copolymer to adopt a compact globular conformation, densifying the interfacial film and enhancing its mechanical strength. Concurrently, the collapse brings the ionized sulfonate groups of AMPS into closer proximity at the interface, increasing the surface charge density (zeta potential increased from – 32.5 mV to – 39.4 mV upon thermal aging) and providing sustained electrostatic repulsion. This synergistic combination of steric reinforcement and electrostatic stabilization enables the PSNA-2-based OBDF to withstand the increased interfacial stress associated with the low OWR, a capability not observed in the nonthermoresponsive control copolymer (PSA) or in the commercial emulsifiers tested. The environmental compatibility of the PSNA-2 formulation was confirmed by an EC_50_ value of 36,860 mg/L, exceeding the 30,000 mg/L nontoxic threshold, highlighting its potential as an environmentally friendly alternative to conventional emulsifiers.

Despite the robust indirect evidence provided for the thermally triggered self-reinforcement mechanism, we acknowledge that direct in situ visualization of the coil-to-globule transition and the corresponding interfacial film compaction remains unaddressed in the present work. The mechanistic conclusions, while strongly supported by the convergence of multiple independent measurements (LCST, IFT, droplet size, and zeta potential), would benefit from direct interfacial characterization in future studies. (i) direct characterization of the temperature-dependent conformational transition at the oil–water interface using advanced techniques such as interfacial rheology, quartz crystal microbalance with dissipation monitoring (QCM-D), or sum-frequency generation (SFG) spectroscopy; (ii) systematic evaluation of formulation versatility, including compatibility with different base oils (e.g., diesel, synthetic oils) and alternative weighting agents (e.g., ilmenite, Mn_3_O_4_); and (iii) pilot-scale synthesis followed by field trials under actual downhole circulating conditions, particularly in deviated wells at approximately 150 °C and an OWR of 70:30, to validate the practical applicability of the PSNA-2 system. The present work establishes a scalable, cost-effective, and environmentally benign design strategy for adaptive thermoresponsive polymer emulsifiers, offering a promising pathway toward high-performance low-OWR OBDFs for deep and challenging drilling operations.

## Data Availability

The data that support the findings of this study are available from the corresponding author upon reasonable request.
